# In situ analysis of acupuncture protecting dopaminergic neurons from lipid peroxidative damage in mice of Parkinson's disease

**DOI:** 10.1111/cpr.13213

**Published:** 2022-03-11

**Authors:** Tingting Zuo, Mo Xie, Meiling Yan, Zengyan Zhang, Tian Tian, Ying Zhu, Lihua Wang, Yanhong Sun

**Affiliations:** ^1^ Division of Physical Biology, CAS Key Laboratory of Interfacial Physics and Technology Shanghai Institute of Applied Physics, Chinese Academy of Sciences Shanghai China; ^2^ University of Chinese Academy of Sciences Beijing China; ^3^ State Key Laboratory of Organic Electronics and Information Displays & Jiangsu Key Laboratory for Biosensors, Institute of Advanced Materials (IAM), Jiangsu National Synergetic Innovation Center for Advanced Materials (SICAM) Nanjing University of Posts and Telecommunications Nanjing China; ^4^ The Interdisciplinary Research Center, Shanghai Synchrotron Radiation Facility, Zhangjiang Laboratory Shanghai Advanced Research Institute, Chinese Academy of Sciences Shanghai China; ^5^ Institute of Interdisciplinary Integrative Medicine Research Shanghai University of Traditional Chinese Medicine Shanghai China

## Abstract

**Objectives:**

Acupuncture stimulation has proven to protect dopaminergic neurons from oxidative damage in animal models of Parkinson's disease (PD), but it remains unclear about the in situ information of biochemical components in dopaminergic neurons. Here, we aimed to analyse in situ changes of biochemical components and lipid peroxidation levels in dopaminergic neurons in PD mice treated with acupuncture by synchrotron FTIR micro‐spectroscopy technique.

**Materials and Methods:**

About 9–10‐week‐old C57BL/6 mice were used to establish PD model by intraperitoneal injection of 1‐methyl‐4‐phenyl‐1,2,3,6‐tetrahydropyridine (MPTP, 30 mg/kg for 5 days). Acupuncture stimulation was performed once a day for 12 days. Behaviour test was determined using the rotarod instrument. Biochemical compositions of dopaminergic neurons in substantia nigra pars compacta were analysed by synchrotron FTIR micro‐spectroscopy technique. The number and ultrastructure of dopaminergic neurons were respectively observed by immunofluorescence and transmission electron microscopy (TEM).

**Results:**

We found that the number and protein expression of dopaminergic neurons in MPTP‐treated mice were reduced by about half, while that in the mice treated by acupuncture were significantly restored. Acupuncture treatment also restored the motor ability of PD mice. The results of single cell imaging with synchrotron FTIR micro‐spectroscopy technique showed that the proportion of lipid in MPTP treated mice increased significantly. Especially the ratio of CH_2_ asymmetric stretching and CH_3_ asymmetric stretching increased significantly, suggesting that MPTP induced lipid peroxidation damage of dopaminergic neurons. It is also supported by the result of TEM, such as mitochondrial swelling or atrophy, loss of mitochondrial crests and mitochondrial vacuolization. Compared with MPTP treated mice, the proportion of lipid in acupuncture treated mice decreased and the mitochondrial structure was restored.

**Conclusions:**

Acupuncture can inhibit the level of lipid peroxides in dopaminergic neurons and protect neurons from oxidative damage. The study provides a promising method for in situ analysis of biochemical compositions in PD mice and reveals the mechanism of acupuncture in treating neurodegenerative diseases.

## Graphical abstract

Synchrotron FTIR micro‐spectroscopy technique was used to analyse in situ changes of biochemical components and lipid peroxidation levels in dopaminergic neurons in Parkinson's disease (PD) mice treated with acupuncture. Results showed that the lipid proportion increased significantly in MPTP induced PD mice, including the ratio of lipid to protein, the ratio of lipid to nucleic acid and the ratio of CH_2_ asym str to CH_3_ asym str. In particular, the increase of the ratio of CH_2_ asym str to CH_3_ asym str implies the higher level of lipid peroxidation. The acupuncture stimulation at Yanglingquan (GB34) acupoints lowered the lipid proportion in dopaminergic neurons of PD mice brain. Our study successfully assesses acupuncture inhibiting oxidative stress damage in dopaminergic cells by in situ analysis of FTIR micro‐spectroscopy.

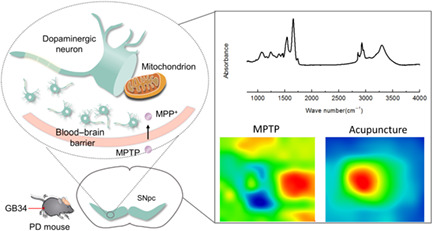

## 1 INTRODUCTION

Parkinson's disease (PD), as a common neurodegenerative disease, is characterized by progressive loss of dopaminergic neurons in the substantia nigra pars compacta (SNpc) of brain.^1^, ^2^ Despite its pathogenesis still remaining unclear, oxidative stress plays an important role in the loss of dopaminergic neurons in PD.^3^, ^4^, ^5^ Since the oxidative metabolism of dopamine produces free radicals, dopaminergic neurons in SNpc are vulnerable to oxidative stress.^6^ Previous studies have shown that the contents of metal elements (e.g., copper, zinc and manganese) in cerebrospinal fluid of patients with PD were significantly higher than those of healthy ones.^7^, ^8^ Moreover, in the brains of PD patients and animal models, the levels of metals in the SNpc were also higher than those of normal control.^9^, ^10^ These abnormal metal elements can cause oxidative damage to dopaminergic neuron cells.^3^, ^4^, ^7^, ^9^ Therefore, antioxidant therapy has become one of the treatments of PD. Some drugs have antioxidant neuroprotective effects, can delay the progress of the symptoms of PD in the experimental study,^11^, ^12^, ^13^, ^14^ but they still have not proven to be clinically safe or effective. Furthermore, these drugs face the challenge of crossing the blood–brain barrier and overcoming systemic side effects.

Acupuncture is a physical therapy in traditional Chinese medicine. It is widely used in analgesia, anti‐inflammatory and the treatment of movement disorders.^15^, ^16^, ^17^, ^18^ In recent years, acupuncture has attracted wide attention due to the in‐depth study of mechanism.^17^, ^18^, ^19^, ^20^ Acupuncture stimulation has proven to protect dopaminergic neurons and improve the motor control ability of PD in experimental and clinical study.^15^, ^21^, ^22^, ^23^ It was reported that acupuncture at Yanglingquan acupoints (GB34) could promote the autophagic clearance of alpha‐synuclein protein to alleviate the symptoms of 1‐methyl‐4‐phenyl‐1,2,3,6‐tetrahydropyridine (MPTP)‐induced PD.^24^ Several studies have shown that acupuncture antioxidative effects in PD mouse model.^25^, ^26^, ^27^, ^28^ Although acupuncture can protect dopaminergic neurons against oxidative damage, it remains unclear about the in situ information of biochemical components in dopaminergic neurons. Hence, we could not know which components of peroxidation were inhibited by acupuncture. The technique for in situ analysis of biochemical components was helpful to analyse the changes of components in SNpc of PD mice and acupuncture‐treated PD mice.

**FIGURE 1:**
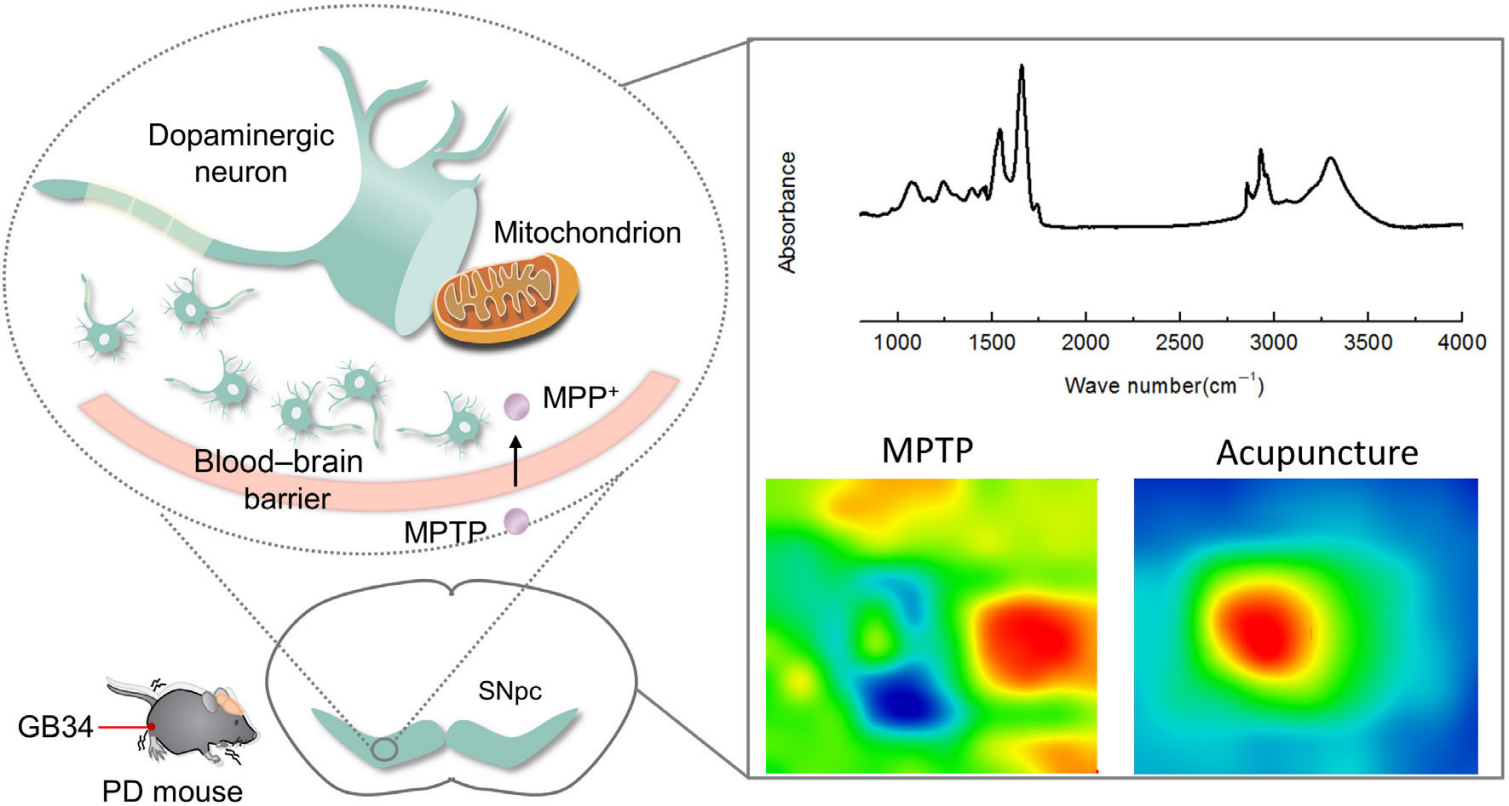
Experimental scheme. Mouse model was established by injecting MPTP. Acupuncture treatment was performed once a day for 12 days. At Day 12, substantia nigra pars compacta (SNpc) of the brain was separated to analyse the components of proteins, lipids and nucleic acid in dopaminergic neurons by synchrotron FTIR micro‐spectroscopy technique

Synchrotron FTIR micro‐spectroscopy technique has become a powerful tool for the analysis of the biochemical composition and structure of biological samples. It combines the high brightness of synchrotron radiation light source with the micro‐imaging function of infrared microscopy.^29^, ^30^ Therefore, high quality infrared spectrum and imaging can be provided with micron‐scale spatial resolution. We can achieve in situ information including the structure, content and distribution of biochemical molecules in biological tissues and cells by synchrotron FTIR micro‐spectroscopy technique. It has been used to study protein aggregation in degenerative diseases.^31^, ^32^, ^33^ Here, we aimed to analyse in situ changes of biochemical components and lipid peroxidation levels in dopaminergic neurons in PD mice treated with acupuncture by synchrotron FTIR micro‐spectroscopy technique (Figure 1). At the same time, the number and ultrastructure of dopaminergic neurons were respectively observed by immunofluorescence staining and transmission electron microscopy (TEM).

## 2 MATERIALS AND METHODS

### 2.1 Animals and treatment

Wild type C57BL/6 mice (male, 9–10 weeks) were obtained from Shanghai SLAC Laboratory Animal Co., Ltd. Mice were housed in animal houses with 22 ± 2°C temperature, 50%–70% humidity and 12 h light/dark cycle. The use and care of animals complied with the guideline of the Ethics Committee of Shanghai University of TCM.

MPTP was used to establish the mouse model of PD. Mice were stochastically distributed to three groups: saline, MPTP and MPTP with acupuncture treatment. The saline group was injected with 0.9% NaCl solution. Mice in the MPTP and acupuncture group were treated by intraperitoneal injection of MPTP‐HCl once a day (30 mg/kg) for 5 days.^34^, ^35^ Mice in the acupuncture group were stimulated with acupuncture at GB34 acupoints once a day for 12 days according to a previous study.^24^ GB34 is an acupoint of the gall bladder meridian, which is located at the depression anterior and inferior to the fibular head of leg. The acupuncture needle (10 mm in length, 0.19 mm in diameter) was inserted to a depth of 3 mm at GB34 acupoints.

### 2.2 Behavioural test

Overall rotarod performances of mice in different groups were determined using rotarod instrument. Before experiment, the mice were trained on the instrument at 20 rpm for 120 s. At Day 12, all mice were trained again on the rod at an accelerating speed from 0 to 30 rpm before testing. Then the mice were tested at 10, 15, 20 and 30 rpm speed for 300 s respectively. Mice were given about 5 min rest between one speed and other speed. The time on the rod of each mice was recorded at every speed. The overall rotarod performance score was calculated according to previous study.^24^

### 2.3 Immunofluorescence staining of dopaminergic neurons in brain

The mice in different groups were perfused with 4% paraformaldehyde after anaesthesia. The brain tissue was stored in 4% paraformaldehyde overnight and dehydrated in gradient sucrose solution from 10% to 30%. The brain tissue containing SNpc was embedded by optimal cutting temperature compound and sectioned at 30 μm thick slices by freezing microtome (Leica CM1950). After washing thrice with PBS, these tissue slices were completely incubated with 3% H_2_O_2_ solution for 15 min to inactive peroxidase in vivo. Next, the sections were incubated with 6% BSA containing 0.25% Triton X‐100 for 60 min. After that, the tissue slices were incubated with tyrosine hydroxylase antibody (Millipore, MAB318, Monoclonal Antibody, 1:400, mouse) overnight at 4°C and then incubated with a secondary antibody of Alexa Fluor® 488 (Abcam, 150113, Polyclonal Antibody, 1:1000, mouse). Finally, the slices were covered by an anti‐quenching agent of antifade mounting medium from Shanghai Beyotime Technology Co., Ltd. The images were obtained by Leica confocal SP8 microscope and Zeiss Imager Axioskop 2. The tyrosine hydroxylase (TH)‐positive cells from a series of sections in the same region of SNpc in the brain were counted and analysed by the Image J software.

### 2.4 Western blotting analysis

The brain tissue containing SNpc was crushed, and homogenized in the 4°C pre‐cooled RIPA lysis buffer. Tissue debris in the lysate was removed by high‐speed centrifugation and the protein concentration was measured by BCA protein assay kit (Thermo Fisher Scientific Inc.). Protein samples were boiled for 5 min in SDS loading buffer and then subjected to 12.5% SDS‐PAGE gel. The target protein bands were transferred to the PVDF membrane, which was blocked for 60 min using Blocking Buffer (P0023B, Beyotime). Then PVDF membrane was incubated with TH antibody (MAB318, Millipore, 1:1000) and GAPDH (1:5000, Cell Signaling) overnight at 4°C. After extensive washing in PBST (PBS containing 0.1% Tween 20) buffer, the blots were probed with an HRP‐labelled IgG (H+L) antibody (Beyotime, 1:10,000, mouse) for 1 h at room temperature. The blots were then incubated with a developing agent of chemiluminescence plus and captured by the SYBR imaging system.

### 2.5 Analysis of metal elements in substantia nigra

The brain slice containing the SNpc (30‐μm thick) was fixed on ~3 μm mylar film. Mental elemental mapping of Cu, Fe and Zn was obtained at BL15U1 beamline of Shanghai Synchrotron Radiation Facility. An X‐ray energy of 10 keV was used to excite the Kα fluorescence of Cu, Fe and Zn. The beam spot of X‐ray was adjusted to 100 μm × 100 μm by narrow slits. The brain slices were raster‐scanned in 100‐μm steps in the *x* and *z* directions. The dwell time was 1 s each pixel and about 4 h for the whole sample. Standard reference materials of bovine liver (NIST 1577a) were scanned under the same experimental conditions as brain slice. Contents of Fe, Cu and Zn in the slices were obtained via the normalization to Compton scattering intensity, then the ratio of Compton intensity against the concentrations of each element in the standard reference materials. Based on the elemental contents, 2D elemental maps with false colour were obtained using Plot2d.py software.

### 2.6 Synchrotron FTIR mapping

The brain tissue containing the SNpc section (30‐μm thick) was placed on 1‐mm thick BaF_2_ windows. The Synchrotron radiation infrared microscopy and imaging were carried out on the beamline BL01B of SSRF.^36^ The IR micro‐spectra were collected in transmission mode using the infrared microscope coupled with the FTIR spectrometer. Choose a perform mapping scan on the appropriate area to obtain the mapping spectrum, set the scan parameters to zoom in the micro‐area, the spectral range is 800–4000 cm^−1^, the resolution is 4 cm^−1^. The red points indicated the area imaged by synchrotron radiation‐based Fourier‐transform infrared spectromicroscopy. The mid‐infrared absorption degree of FTIR‐mapping is expressed by the degree of chromatic aberration, the high absorption area is red, and the low absorption area is green.

### 2.7 Transmission electron microscopy

The mice were perfused transcardially with 4% paraformaldehyde and 1% glutaraldehyde in phosphate buffer, the SNpc of the brain was fixed in 2.5% glutaraldehyde for 2 h. The slices of 100‐μm thick were cut by a vibratome (Leica) and then fixed in 1% osmium tetroxide for 1 h with a temperature of 4°C. The sections were dehydrated and embedded in epoxy resin, then cut into 100‐nm thickness. Finally, the sections were stained with 5% uranyl acetate and examined by TEM.

### 2.8 Statistical analysis

All statistical analyses were performed using GraphPad Prism software version 5 and statistical significance was analysed using one‐way ANOVA test (*p < 0.05, **p < 0.01).

## 3 RESULTS

### 3.1 Acupuncture inhibits the loss of dopaminergic neurons in MPTP‐induced PD mice

**FIGURE 2:**
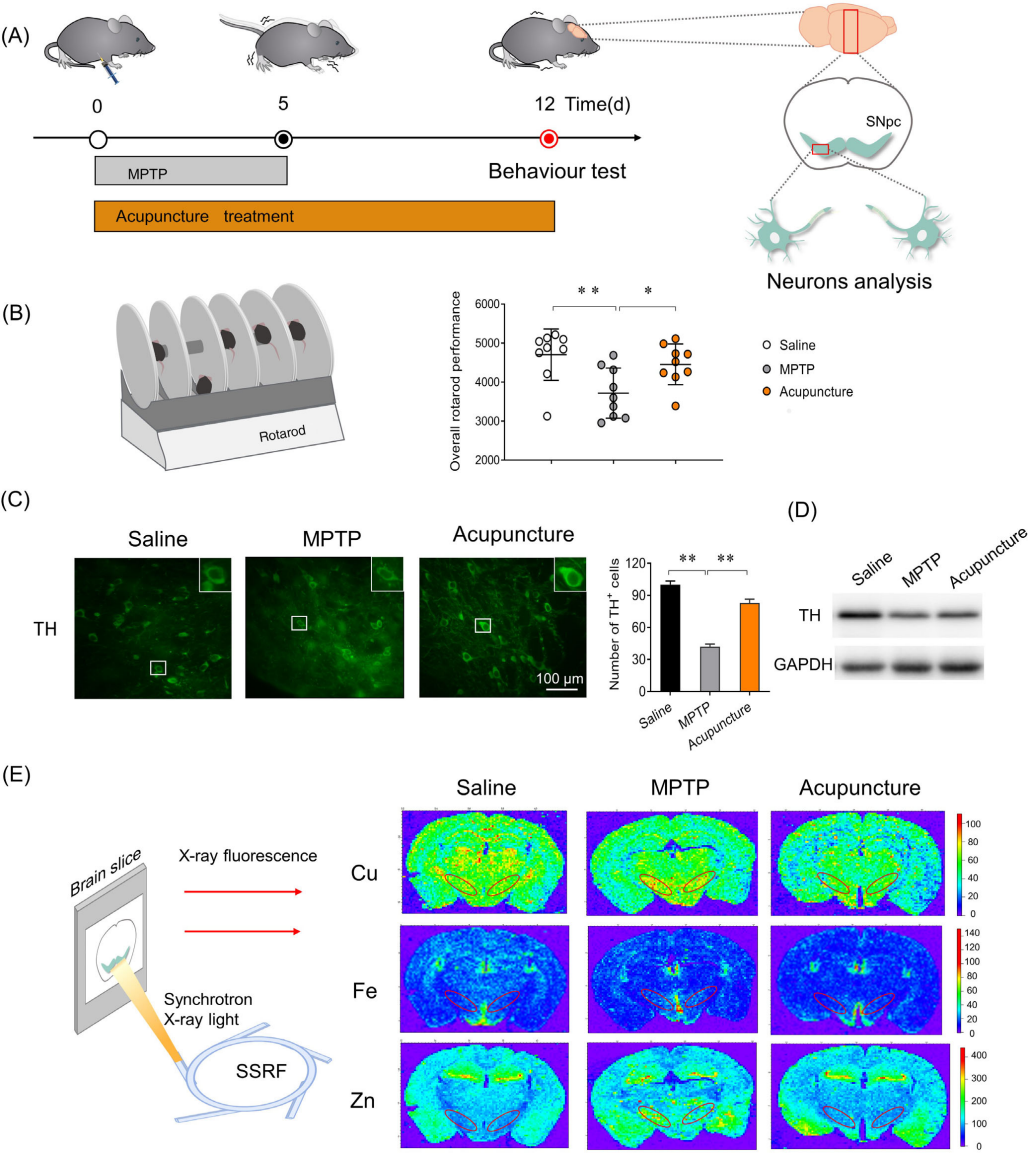
Acupuncture inhibits the loss of dopaminergic neurons in SNpc. (A) Schematic of PD model preparation, acupuncture treatment, behaviour test and dopaminergic neuron analysis. (B) Overall rotarod performance score of mice in different groups (*n* = 9). (C) Immunofluorescence images and the relative number of TH‐positive cells in substantia nigra pars compacta (SNpc) in each group. Scale bar, 100 μm. (D) Protein expression analysis of TH by western blot. (E) Elemental mapping of Cu, Fe and Zn in brain slices of mice in different groups. The region in the red ellipse is the SNpc section. In all panels, results were presented as mean ± SD, one‐way ANOVA test was used to analyse the difference among the groups. *p < 0.05, **p < 0.01 compared with MPTP group

We first observed the inhibiting effect of acupuncture on loss of dopaminergic neurons of MPTP induced PD mice (Figure 2A). TH, a marker protein of dopaminergic neurons, was respectively analysed by western blot and immunofluorescence staining. Approximately 9‐week‐old mice were used to establish the MPTP‐induced PD model according to previous studies. Two hours after the first injection of MPTP, acupuncture stimulation at GB34 was performed once a day for 12 days. At the end of the experiment, the behaviour was determined using rotarod instrument. Results were shown in Figure 2B, the overall rotarod performance scores of MPTP treated mice (3717 ± 644.1) were about 20% lower than those of the saline group (4702 ± 661.2), there was significant difference between saline group and MPTP group (Figure 2B, *p* < 0.01), implying the symptom of PD in mice of MPTP group. The mice treated by acupuncture (4455 ± 522.6) obtained excellent scores compared with mice in MPTP group (*p* < 0.05). Result indicated acupuncture promoted the motor function of PD mice.

Next, the SNpc of the brain was isolated for protein expression analysis of TH by western blot. The number of TH‐positive cells in SNpc was detected by immunofluorescence staining in ~30 μm brain slices. We found the number of TH‐positive cells (Figure 2C) and the expression of TH protein (Figure 2D) in MPTP‐induced PD mice decreased by 50%–60% compared with saline treated mice, suggesting that PD mouse model is established successfully. While in the acupuncture treated mice, the number of TH‐positive cells is about twice that of the model group, which is 83% of the saline group. The expression of TH protein in the acupuncture group also increased twice of the PD group, which is 75% of the saline group. These results indicated that acupuncture effectively inhibited loss of dopaminergic neurons induced by MPTP. As we know, metal elements play an important role in the course of neural activity. But excessive metal concentration is involved in the oxidative damage to neuron cells.^3^, ^4^ In this study, we found the metal elements of Cu, Fe and Zn in PD mice induced by MPTP were all higher than those of saline group in the SNpc section (Figure 2E), which may be related to the loss of dopaminergic neurons. After acupuncture treatment, the metal elements of Cu, Fe and Zn obviously decreased in the SNpc of brain, suggesting that acupuncture could regulate the distribution and the concentration of metal elements in the brain.

### 3.2 Acupuncture protects the dopaminergic neurons from lipid peroxidative damage

**FIGURE 3:**
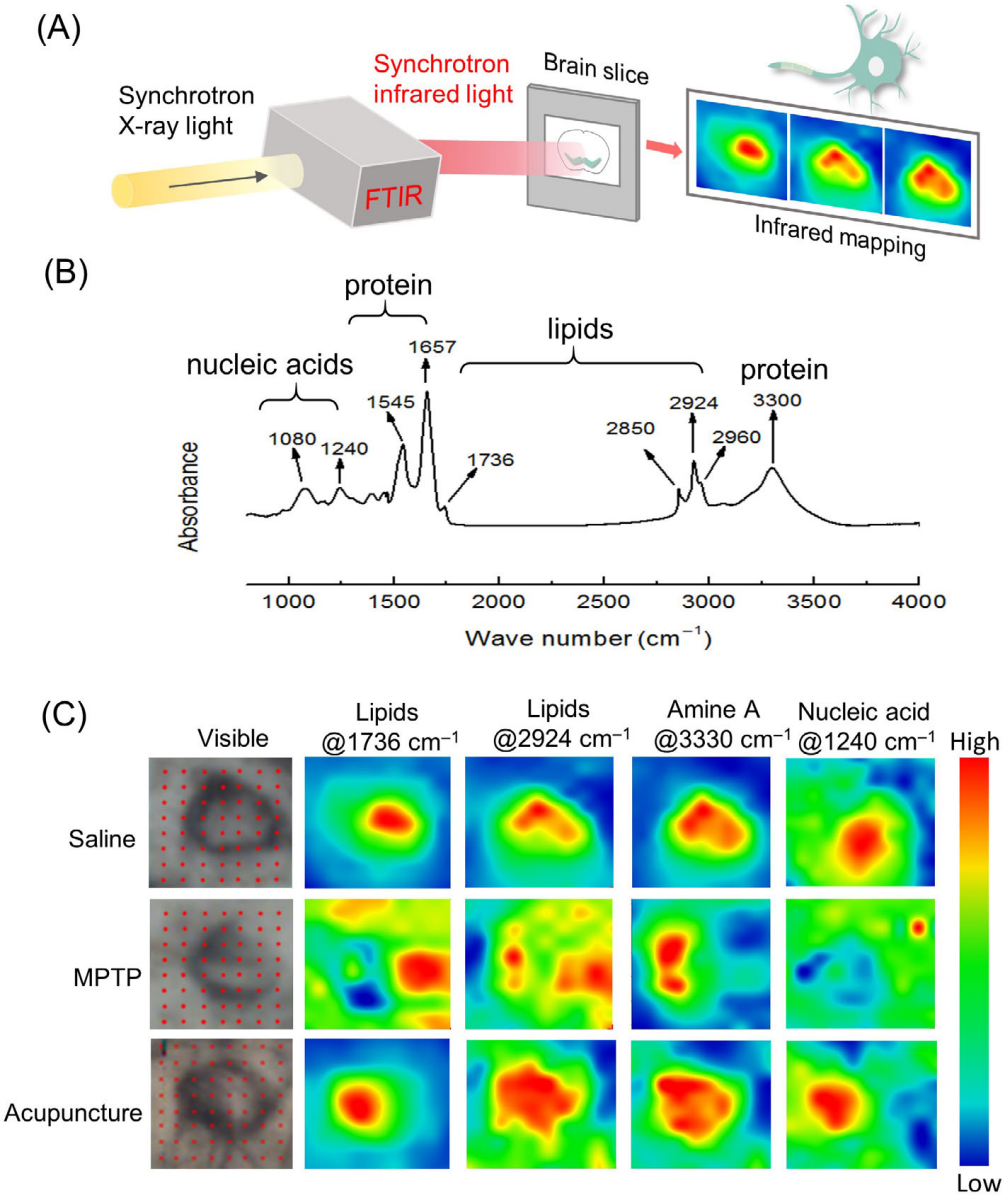
Synchrotron radiation FTIR imaging of dopaminergic neuron in SNpc. (A) Schematic showing of Synchrotron FTIR mapping by SSRF. (B) FTIR absorption spectra acquired in the neurons of brain sample. (C) The intracellular biochemical components by synchrotron radiation FTIR micro‐spectroscopy technique

Based on the results that acupuncture inhibited the loss of TH‐positive cells, we further analysed the intracellular biochemical components by synchrotron radiation FTIR micro‐spectroscopy technique (Figure 3A). The different components in cells have characteristic infrared absorption peaks (Figure 3B and Table S1). The spectral absorption features for lipids were found at 1736, 2850, 2924 and 2960 cm^−1^, which increased in neuron cells of MPTP mice and distributed throughout the cell (Figure 3C). Compared with MPTP mice, the lipid in acupuncture‐treated group was decreased obviously. The spectral absorption features for proteins were found at 1657, 1545 and 3330 cm^−1^, there was a decrease in the level of amide between saline group and MPTP group, and acupuncture increased the amide level of PD mice. We could find the spectral absorption features for nucleic acid at 1080 and 1240 cm^−1^, there were no obvious changes in quantity among different groups (Figure 3C). However, the distribution of nucleic acid was different from saline and acupuncture groups, which seemed to escape from the cell. These results indicate that MPTP causes an increase in lipid levels in neuron cells to induce cell damage and cellular component spillover from the cells, simultaneously, acupuncture lowers the lipid levels and increases the amide levels of dopaminergic neurons in SNpc section. The abnormal distribution of nucleic acid was rescued by acupuncture (Figure 3C).

**FIGURE 4:**
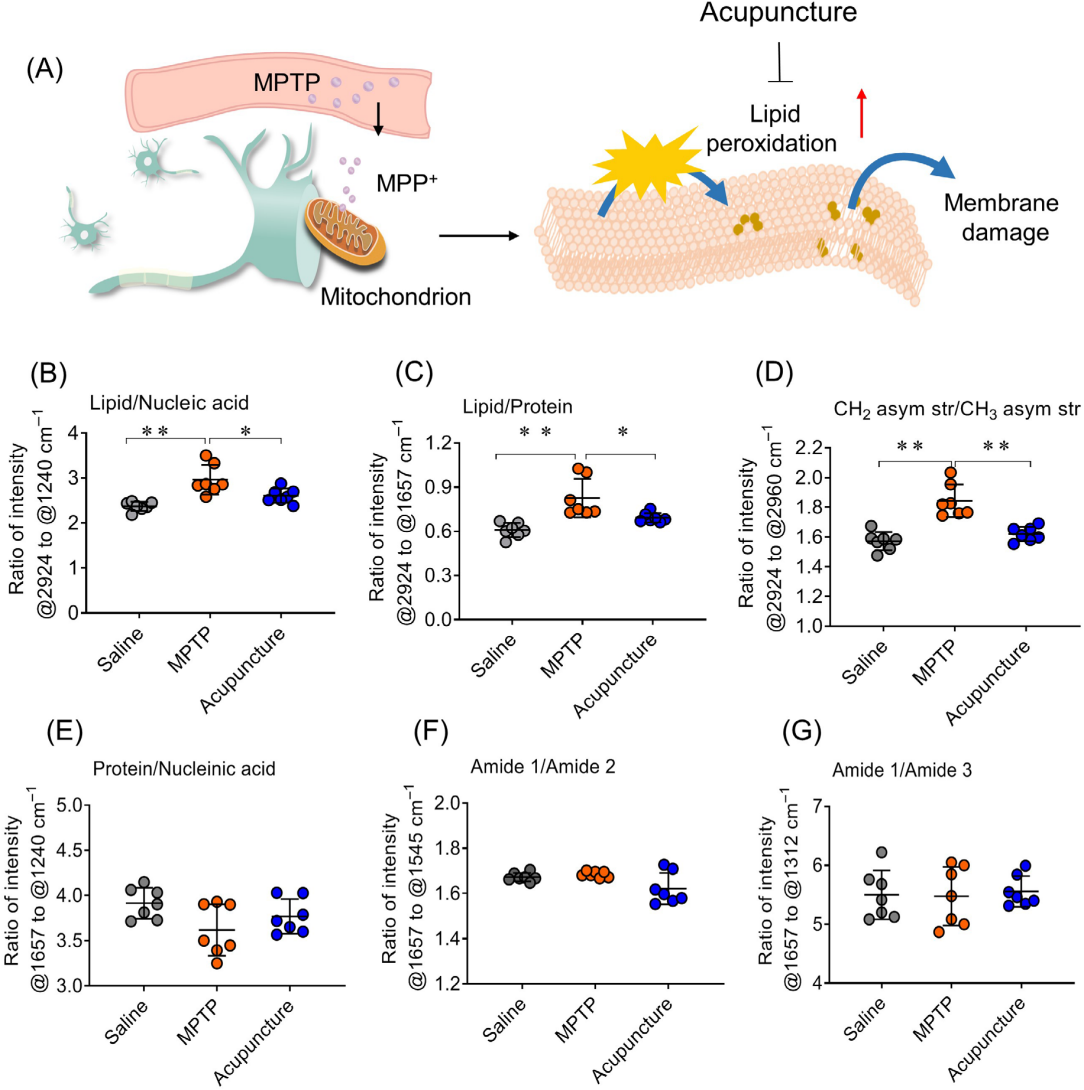
Relative intensity of protein, lipid and nucleic acid in dopaminergic neurons of different groups. (A) MPTP‐induced oxidative damage of neuron cells. (B) Ratio of lipid @2924 cm^−1^ to nucleic acid @1240 cm^−1^. (C) Ratio of lipid (@2924 cm^−1^ to protein @1657 cm^−1^. (D) Ratio of CH_2_ asym str @2924 cm^−1^ to CH_3_ asym str @2960 cm^−1^. (E) Ratio of protein @1657 cm^−1^ to nucleic acid @1240 cm^−1^. (F) Ratio of amide 1 @1657 cm^−1^ to amide 2 @1545 cm^−1^. (G) Ratio of amide 1 @1657 cm^−1^ to amide 3 @1312 cm^−1^. One‐way ANOVA test was used to compare the difference among the groups. *n* = 7, *p < 0.05, **p < 0.01

To further analyse relative proportions of proteins, lipids and amino acids in the cells, we scanned multiple cells in the sample of different groups. MPTP was dehydrogenated to MPP^+^ ions in the cells of SNpc, which inhibit mitochondrial complex enzymes. As a result, accumulation of lipid peroxidation products leads to damage of neuron cells (Figure 4A). From Figure 4B–G, we found the ratio of lipid to nucleic acid and the ratio of lipid to protein were higher than those of the control group (Figure 4B,C, *p* < 0.01). Furthermore, the ratio of CH_2_ asymmetric stretching (CH_2_ asym str) to CH_3_ asymmetric stretching (CH_3_ asym str) significantly increased in the cells of SNpc compared with control group (Figure 4D, *p* < 0.01), implicating the low level of the unsaturated phospholipids and high level of lipid peroxidation due to oxidation stress.^31^, ^37^ After acupuncture treatment, the ratio of lipid to nucleic acid and the ratio of lipid to protein were lower than those of the MPTP group. In particular, the ratio of CH_2_ asym str to CH_3_ asym str appeared a significant decrease in the acupuncture group compared with the model group (*p* < 0.01), suggesting acupuncture could reduce the level of peroxidation and the content of lipid to alleviate the oxidative stress damage caused by MPTP. In addition, it was found there were no obvious differences in the ratio of protein to nucleic acid (Figure 4E). There was no significant change in the level and structure of amides including the ratio of amide 1 to amide 2 and amide 1 to amide 3 among different groups (Figure 4F,G). Through the mapping and spectroscopic analysis of certain components (lipid, protein and nucleic acid) in neuron cell by synchrotron radiation FTIR, we not only obtained the information of the content and distribution of oxidative damage components in PD mice, but also understood the in situ information of acupuncture against oxidative damage of neuronal cells.

### 3.3 The effect of acupuncture on ultrastructure of dopaminergic neurons

**FIGURE 5:**
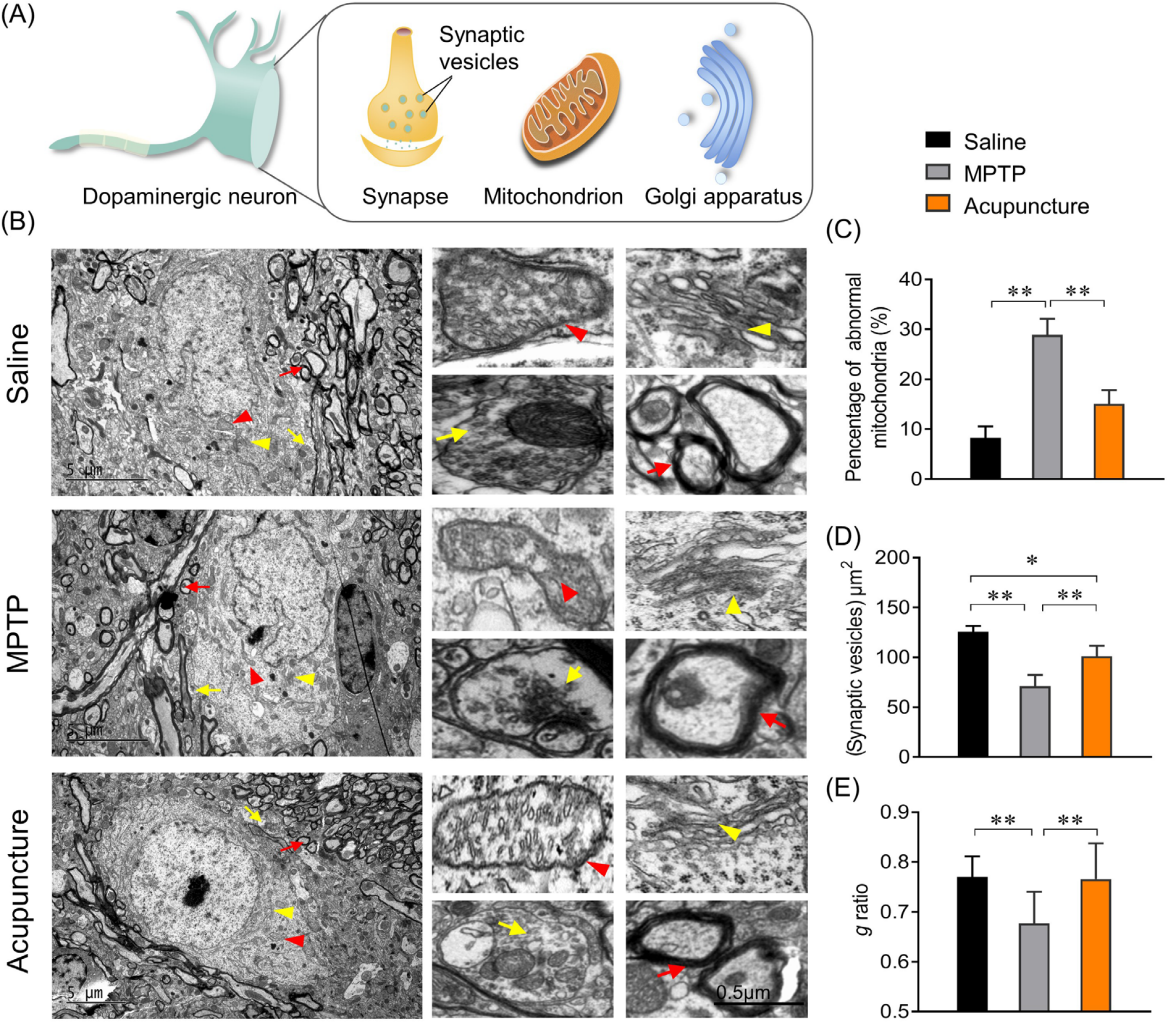
Effect of acupuncture on ultrastructure of neuron cells in substantia nigra pars compacta (SNpc). (A) Schematic diagram of the organelles in neuron cells. (B) Representative electron micrographs of neurons cells in SNpc. Red triangle, mitochondria; yellow triangle, golgi apparatus; red arrow, axon; yellow arrow, synaptic vesicles . Scale bar, 5 μm (left), 0.5 μm (right). (C) The analysis of abnormal mitochondria. (D) Analysis of synaptic vesicle density in the presynaptic bouton area. (E) *g* ratio analysis of axon in SNpc. In all panels, *n* = 4, results were presented as mean ± SD, one‐way ANOVA test was used to analyse the difference among the groups. *p < 0.05, **p < 0.01

Finally, we investigated the ultrastructure of dopaminergic neurons by TEM (Figure 5A,B). Mitochondria, the crucial organelles, are the main place of oxidative phosphorylation to provide energy for cells, which are known as the ‘powerhouse’ of cells. Pathologically, mitochondria are the most vulnerable to oxidative damage, resulting in mitochondrial structural changes, such as mitochondrial swelling or shrinkage, loss of mitochondrial crests and mitochondrial vacuolization. In the saline group, the structure of mitochondria and Golgi apparatus were intact, the mitochondria structure in axons was clear (Figure 5B). While in the MPTP group, mitochondrial membrane was incomplete, the density of mitochondrial matrix and cristae decreased significantly. The horseshoe‐shaped structures of Golgi apparatus were damaged, exhibiting smaller lacunae and less folding. Vesicles in the dendrites were reduced and the myelin sheaths of the axons were disorganized. From further analysis of the images of TEM, we found that the proportion of abnormal mitochondria in MPTP‐induced PD mice was about twice that of mice in the saline group (*p* < 0.01). While in the acupuncture treated mice, the abnormal mitochondria is about half of that in the model group mice (Figure 5C, *p* < 0.01), implying that acupuncture treatment can inhibit the mitochondrial damage induced by MPTP. The density of synaptic vesicles in saline group mice was 125.75 ± 5.82/μm^2^, but that in MPTP‐induced PD mice reduced to 71.05 ± 9.91/μm^2^, there was significant difference between them (*p* < 0.01). The *g* ratio, as a function of axonal diameter analysis, was decreased in the MPTP‐induced PD mice than saline treated mice, suggesting that the thickness of myelin in PD mice is increased to affect the conduction of impulses. After acupuncture treatment, the density of synaptic vesicles was increased to 101.20 ± 5.82/μm^2^ and the *g* ratio was elevated significantly (Figure 5D,E, *p* < 0.01). These results suggest that MPTP damaged the ultrastructure of dopaminergic neurons seriously. After acupuncture treatment, the structure of the organelles was rescued. Especially, the envelope of the mitochondria was intact, the structure of mitochondrial cristae was restored, which improved the function of synapse and axon.

## 4 DISCUSSION

Oxidative stress plays an important role in the loss of dopaminergic neurons in PD.^3^, ^4^, ^5^ Since the oxidative metabolism of dopamine produces free radicals, dopaminergic neurons in SNpc are vulnerable to oxidative stress.^6^ In addition, the contents of metal elements (e.g., iron, copper, zinc and manganese) in cerebrospinal fluid and brain of patients with PD are much higher than normal ones. Excessive metal concentration involved in the oxidative damage to neuron cells,^3^, ^4^ particularly iron ions, which react with hydrogen peroxide to generate hydroxyl radicals.^38^ The increase of free radicals generates lipid peroxidation, which causes the member damage and cell degeneration. Hence, lipid peroxidation is frequently used to measure oxidative stress level. Its content not only reflects the degree of oxidative damage, but also can be used to assess the drug of antioxidant damage. In previous study, plasma was used to measure the level of lipid peroxidation, which is higher in PD patients than that of healthy individuals.^39^, ^40^ However, we cannot learn about the in situ information of oxidative stress in neuron cells from the level of lipid peroxidation in plasma. In the present study, we used the FTIR micro‐spectroscopy technique to analyse the components of lipid, protein and nucleic acid in dopaminergic cells of PD mice. Results demonstrate that the lipid proportion increases significantly in MPTP induced PD mice, including the ratio of lipid to protein, the ratio of lipid to nucleic acid and the ratio of CH_2_ asym str to CH_3_ asym str (Figure 4B–D). In particular, the increase of the ratio of CH_2_ asym str to CH_3_ asym str implies the higher level of lipid peroxidation. The components of protein and nucleic acid have no obvious changes. Interestingly, the acupuncture stimulation at GB34 in legs lowered the lipid proportion in dopaminergic neurons of PD mice brain. Results indicate that acupuncture inhibits the generation of lipid peroxidation. Our study successfully assesses acupuncture inhibiting oxidative stress damage in dopaminergic cells by in situ analysis of FTIR micro‐spectroscopy. This work is a preliminary attempt to evaluate the anti‐oxidative damage of acupuncture with synchrotron radiation FTIR micro‐spectroscopy technique, and the detailed mechanism needs to be further investigated.

In addition, synchrotron FTIR micro‐spectroscopy technique combines the high brightness of synchrotron radiation light source with the micro‐imaging function of infrared microscopy.^29^, ^30^ High quality infrared spectrum and imaging can be obtained with micro‐scale spatial resolution. It is a powerful tool for the analysis of the biochemical composition and structure of biological samples. Most importantly, no staining is required when you analyse the samples, the component mappings of proteins, lipids, amides and nucleic acids can be detected at the same time. Other tissues can also be analysed by FTIR micro‐spectroscopy technique, such as tumours, bones, muscles and so on. It provides an intact, in situ mapping method for biological samples analysis.

## CONFLICT OF INTEREST

All authors have no financial disclosures or conflicts of interest to declare.

## AUTHOR CONTRIBUTIONS

Yanhong Sun and Zengyan Zhang designed the study. Tingting Zuo, Mo Xie, Zengyan Zhang, Tian Tian and Meiling Yan performed most of the experiments. Meiling Yan, Tingting Zuo, Zengyan Zhang and Yanhong Sun performed the data analysis for the study. Lihua Wang and Ying Zhu supervised the study. Yanhong Sun, Mo Xie, and Tingting Zuo wrote the paper.

## Supporting information


**Table S1** Tentative assignments of the band's frequencies.

## Data Availability

The data that support the findings of this study are available from the corresponding author upon reasonable request.
