# Supplementary material for: In situ analysis of acupuncture protecting dopaminergic neurons from lipid peroxidative damage in mice of Parkinson's disease
Source: Cell Prolif. 2022 Mar 11;55(4):e13213. doi: 10.1111/cpr.13213 (PMC9055900; doi:10.1111/cpr.13213)
Supplement: Supplementary file 1 — Table S1 Tentative assignments of the band's frequencies. [file CPR-55-e13213-s001.docx]

**Supplementary Information**

**In situ analysis of acupuncture protecting dopaminergic neurons from lipid peroxidative damage in mice of Parkinson's disease**

Tingting Zuo^1,5^, Mo Xie ^3^, Meiling Yan^1,5^, Zengyan Zhang^2^*, Tian Tian^4^, Ying Zhu^1,2^, Lihua Wang^1,2^,Yanhong Sun^1,2^*

1. *Division of Physical Biology, CAS Key Laboratory of Interfacial Physics and Technology, Shanghai Institute of Applied Physics, Chinese Academy of Sciences, Shanghai 201800*
2. *the Interdisciplinary Research Center, Shanghai Synchrotron Radiation Facility, Zhangjiang Laboratory,*

*Shanghai Advanced Research Institute, Chinese Academy of Sciences, Shanghai 201210, China*

1. *State Key Laboratory of Organic Electronics and Information Displays & Jiangsu Key Laboratory for Biosensors, Institute of Advanced Materials (IAM), Jiangsu National Synergetic Innovation Center for Advanced Materials (SICAM), Nanjing University of Posts and Telecommunications, Nanjing 210023, China.*
2. *Institute of Interdisciplinary Integrative Medicine Research, Shanghai University of Traditional Chinese Medicine, Shanghai 201203, China*
3. *University of Chinese Academy of Sciences, Beijing 100049*

Tingting Zuo, Mo Xie and Meiling Yan equally contributed to this work

*Corresponding author, [zhangzengyan@zjlab.org.cn](mailto:zhangzengyan@zjlab.org.cn%20), sunyanhong@sinap.ac.cn

Table 1 Tentative assignments of the band’s frequencies

| Frequency (cm^-1^) | Assignment |
| --- | --- |
| 3330  2924  2960 | Amide A: N-H str (protein)  CH_2_ asym str (lipids)  CH_3_ asym str (lipids) |
| 2850 | CH_2_ sym str (lipids) |
| 1736 | C@O str (ester, lipids) |
| 1657 | Amide1: CO str, CN str, NH bend vib (protein) |
| 1545 | Amide2: NH bend vib, CN str (protein) |
| 1240 | PO_2_ asym str (nucleic aids) |
| 1080 | PO_2_ sym str (nucleic aids) |
